# Expression profiles of miRNAs in giant cell tumor of bone showed miR‐187‐5p and miR‐1323 can regulate biological functions through inhibiting FRS2

**DOI:** 10.1002/cam4.2853

**Published:** 2020-03-10

**Authors:** Yuanhan Jin, Jing Zhang, Hao Zhu, Gentao Fan, Guangxin Zhou

**Affiliations:** ^1^ Department of Orthopedics Jinling Hospital Medical school of Southeast University Nanjing China; ^2^ Department of Orthopedics Jinling Hospital Nanjing Medical University Nanjing China; ^3^ Department of Orthopedics Jinling Hospital Nanjing University Nanjing China

**Keywords:** expression profiles, functions, GCTB, microRNAs

## Abstract

**Background:**

Giant cell tumor of bone (GCTB) is considered to be a kind of borderline tumor, which has a tendency to recur and translocate. MicroRNAs are one type of small noncoding RNA, which can inhibit the translation of targeted mRNA through RNA‐induced silencing complex.

**Methods:**

Microarray was conducted on three groups of tumor tissues and normal tissues from patients with GCTB, and results showed different expression profiles of miRNAs with Gene Ontology analysis and Kyoto Encyclopedia of Genes and Genomes analysis. The functions of miR‐187‐5p and miR‐1323, which were highly expressed in GCTB, were examined by 5‐ethynyl‐2′‐deoxyuridine (EDU), transwell, and CCK8 assays. RNAhybrid et al. (rna prediction softwares) predicted that the two microRNAs targeted fibroblast growth factor receptor substrate 2 (FRS2), which was verified by luciferase assay and rescue experiments.

**Results:**

miR‐187‐5p and miR‐1323 were highly expressed in tumor tissues. They can jointly regulate the biological functions of GCTB in vitro. Luciferase assay confirmed that the two microRNAs can bind to the 3′ untranslated regions (UTR) of mRNA of FRS2. And, rescue experiments verified the relationships between the two microRNAs and FRS2.

**Conclusion:**

There were some different‐expressed microRNAs between GCTB and normal tissues. miR‐187‐5p and miR‐1323 can regulate the biological functions of GCTB through influencing the expression of FRS2.

## Graphical abstract

Our team had performed the microarrays on three groups of giant cell tumors of bone. And we found some aberrant expression of microRNAs, including miR‐187‐5p and miR‐1323. Then, we examined the two miRNAs' effects on giant cell tumor of bone.

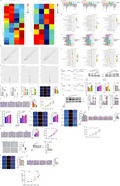

## 1 INTRODUCTION

Giant cell tumor of bone (GCTB) is a type of borderline tumor.^1^, ^2^ It has low morbidity but high recurrence, so well as high metastasis rate, especially translocating to the lung. Giant cell tumor of bone can occur in most long bones in human bodies, including the distal femur and proximal tibia. And, people aged from 20 to 40 have more morbidity than other age groups.^3^ The main pathological ingredients of GCTB include multinucleated giant cells and stromal cells, which have the ultrastructure of osteosarcoma. Meanwhile, the high expression of Receptor activator for nuclear factor‐κ B ligand (RANKL) has significant meaning in the development and treatment of GCTB.^4^ At present, the main therapies include surgery and medicine.^5^ Although the permission of Denosumab (monoclonal antibody for RANKL) treatment on patients with GCTB has been realized,^5^ the recurrence and metastasis of GCTB still need other measures to control. Much more molecules and cytokines' functions in GCTB need more exploration.

MicroRNAs are one kind of small noncoding RNAs with the length of 19‐25 nt,^6^ which can bind to the 3′UTR of mRNA with Argonaute proteins to form RISC (RNA‐induced silencing complex), resulting in the inhibited expression of mRNA.^7^ Bussing et al firstly found that let‐7 is a highly conserved miRNA, which can influence many pathways and processes in cancers.^8^ After that, many researches on the mechanism of microRNAs regulating the occurrence and development of tumors have sprung up.^9^, ^10^ There have been some miRNAs studied on GCTB, such as miR‐30a,^11^ miR‐127,^12^ and miR‐376a.^12^ These miRNAs could regulate the development of GCTB. However, there are few researches showing a systematic expression profile on microRNAs in GCTB.

This study used microarrays on tissues from patients of GCTB to show the different expression profiles of microRNAs. Moreover, with the detection of microRNAs, we analyzed them through GO (Gene Ontology) analysis and KEGG (Kyoto Encyclopedia of Genes and Genomes) analysis to explore and summarize the functions of these microRNAs deeply. At the same time, we found miR‐187‐5p and miR‐1323 had higher expression in tumor tissues than normal tissues. Thus, we studied the two RNAs about their functions in GCTB in vitro. The aims of the research were to investigate more miRNAs functioning in GCTB and provide some usable information for the treatment of GCTB in the future.

## 2 METHODS AND MATERIALS

### 2.1 Specimens Collection

Specimens were collected from the patients with GCTB (information shown in Table S1) in Jinling Hospital (Nanjing, China) from January 2016 to January 2019. While the tumor tissues were cut from the patient's bodied, they were put in the collagenase B (Roche Diagnostics). After the primary culture of GCTB cell line, the redundant tissues were stored in refrigerator at −80°C. At the same time, part of normal tissues, which were adjacent to the tumor tissues, was cut and stored at the same state.

The research has been approved by the Ethics Committee of Jinling Hospital (Nanjing, China). And, the use of patients' tissues for the research has been permitted by patients with their signature on the consent files.

### 2.2 Cell culture and transfection

The cell line (GCTB) was established from the tumor samples of patients. The tissues were cut into small pieces and digestion of 1.5 mg/mL collagenase B for 3 hours at 37°C in Dulbecco's Modified Eagle Medium (DMEM; Gibco),which contained 10% fetal beef serum (FBS; Gibco) and 100 U/mL penicillin/streptomycin (Gibco). Then, cells suspension was centrifuged, washed twice with phosphate buffer liquid, and cultured in the conditional medium. Twenty‐four hours later, the cells were treated with Trypsin (Gibco) to suspend the cells. After three passages, detached cells were cultured to eliminate any remaining giant cells. The main cells we cultured were stromal cells, confirmed by the immunofluorescence of CD68 and PCNA (Figures [Link], [Link], [Link]). When the density of cells grew to 80%, we transfected the microRNA mimics and inhibitors into the cells with Lipofectamine^®^ 3000 (Invitrogen). The transfected concentrations of microRNA mimics, inhibitors, and plasmids were dependent on the situations and expectations.

### 2.3 Microarray analysis

Microarray was conducted with the Affymetrix^®^ GeneChip miRNA array analysis (Affymetrix), provided by Shanghai Biotechnology Corporation, to explore the expression profiles of microRNAs in GCTB and adjacent normal tissues. The total RNA was extracted from tissues through TRIZOL (Invitrogen). Hybridization signals were detected using an Affymetrix^®^ scanner. With the Gene Clustering 3.0 and Java Tree View, the raw data were analyzed.

### 2.4 quantitate Reverse Transcription‐Polymerase Chain Reaction (qRT‐PCR)

The total RNAs were extracted from tissues and cells through TRIZOL. The extracted RNAs were dissolved in Diethypyrocarbonate (DEPC) water. According to the RNA reverse transcription systems, the reverse transcription solution was prepared (Takara). The PCR machines (MiniAmp; Thermo Fisher) were used to complete the reverse transcription process, and cDNAs were obtained. After that, the real‐time quantitative PCR systems (Takara) were prepared according to different cDNAs, and the reactions progressed in the 7900HT Fast Real‐Time PCR System (Life Technologies Corporation). miRNAs and mRNAs were performed with probe methods and SYBR GREEN method, respectively. After the reaction, the instrument can analyze the Ct values according to the signals. Through the threshold, targeted RNAs and glyceraldehyde‐3‐phosphate dehydrogenase (GAPDH)/U6 (internal reference) had their own Ct values. And the deviation between the two Ct values can be considered as ΔCt. The relative RNA levels were represented by 2^−△Ct^. The sequences of the primers of fibroblast growth factor (FGF) receptor substrate 2 (FRS2) are shown in supplementary (Table S2).

### 2.5 Western Blot

The total proteins were extracted from tissues and cells through RIPA. The superior structure of the protein was disrupted with 5× sodium dodecyl sulfate (SDS) solution. The gels were prepared with a concentration of 12.5%. The antibodies we used in the experiments were “mouse anti‐human FRS2” (Santa Cruz, USA), “mouse anti‐human GAPDH” (Santa Cruz, USA), and “goat anti‐mouse” (Santa Cruz, USA). All the procedures followed the manufactures' instructions. The total protein‐SDS solution was added to the gel according to the estimated content of the target protein, and constant voltage of 80 V was applied for electrophoresis with the help of the electrophoresis apparatus (EPS‐300‐IIV; CBS). After the separation of the protein, the gel was taken out and placed in the transfer capsules with a sponge‐thick filter‐gel‐PVDF membrane‐filter paper structure. And, constant current of 0.3 A was applied to transfer the film for 90 minutes. After taking out of the PVDF membranes, according to the ladder, the corresponding bands containing the internal reference protein GAPDH and the target protein on the membranes were cut out. After blocking for 1 hour with 5% milk, the bands were immersed in the respective first antibody solution overnight. Then, the bands were washed by 1× Tris‐HCL+Tween buffer solution (TBST) for several times. And, the bands were incubated with the secondary antibody for 1 hour, followed by TBST washing for several times. Then, the bands were reacted with enhanced chemiluminescent (SuperSignal West Femto; Thermo Fisher) and examined by chemiluminescence detector (Fluoroskan; Thermo Fisher). The instrument can collect the signals from the chemiluminescent and the product of intensities and areas could be considered as the parameters of tested proteins. These were recorded and calculated by ImageJ (NIH). The ratio of the parameters of targeted protein and GAPDH (control) represented the relative level of the targeted proteins.

### 2.6 luciferase reporter assay

Fibroblast growth factor receptor substrate 2 3′UTR was amplified by the cDNA coding FRS2 (NM_006654.5). The pGL3‐basic vectors (Promega) were used to construct the luciferase reporter plasmids, including “wild type” and “mutant type”. These plasmids were co‐transfected with microRNA mimics into the cells we cultured. After 24 hours, cell lysis products were extracted and the luciferase activity was examined with the dual‐luciferase assay system (Promega). The luciferase activity could represent the binding efficiency of the miRNAs and sequences of 3′UTR. Renilla luciferase activity was used to normalize for transfection efficiency.

### 2.7 EUD assay

5‐Ethynyl‐2’‐deoxyuridine (EDU) assay was conducted with Cell‐LightTM EdU Apollo567 kit (Guangzhou Ruibo Biotechnology). When the density of cells turned to 80%, the mimics or inhibitors were transfected into cells. After 24 hours, reagent A was added to the medium and cells were cultured for another 4 hours. Then, according to the manufacturer's instructions, the cells were stained. When the staining was done, photos were taken under the microscope (EVOS M7000; Thermo Fisher) at 200×. The uracil could emit red fluorescence under green excitation light and the nucleus could emit blue fluorescence under light blue excitation light. When focusing on one field, the numbers of red and blue were counted and calculated with the help of ImageJ (NIH). The relative proliferation rate was represented by the ratio of red and blue.

### 2.8 Transwell assay

The Transwell chambers were bought from CORNING. After transfection, cells were diluted into the concentration of 10^5^/well and translocated to the chambers, which were added with DMEM (free FBS). Under the chambers, DMEM containing 20% FBS was filled in the wells. After culturing for 36 hours, the medium was discarded and cells were stained by crystal violet, presenting with blue. Then, the stained cells were captured under the microscope (EVOS M7000; Thermo Fisher) at 100×. Focusing on one field, the blue spots represented the cells that traversed through the porous membrane and glue to reflect the relative invasion rate. The numbers of blue spots were counted and calculated by ImageJ (NIH).

### 2.9 Cell viability assay

CCK8 reaction mixture (APExBIO) was used to perform the cell viability experiments. About 100 µL of cell suspension from each well was added into a tube containing 6‐mL condition medium to dilute the concentration. Then, the new suspension was averagely spread into 96‐well plates with 100 µL of each well. After culturing for 24, 48, 72, 96, 120 hours, respectively, 10‐µL CCK8 reaction mixture was added to each well. The enzyme‐labeled instrument (Multiskan GO; Thermo Fisher) was used to detect the absorbance of OD450 in each well after culturing the mixture of CCK8 reaction and cells for 2 hours. Every time point had an absorbance which could represent the cellular viability in that time and then comparison of the trend of the absorbance of each sample was the assay result.

### 2.10 Statistical analysis

All data statistics were performed with SPSS 20.0 (IBM). Data are expressed as mean ± SD. One‐way ANOVA analysis was used to compare the differences among groups. *, **, ***, and **** indicate *P* < .05, *P* < .01, *P* < .001, and *P* < .0001, respectively.

## 3 RESULTS

### 3.1 Expression profiles of microRNAs in GCTB

**Figure 1:**
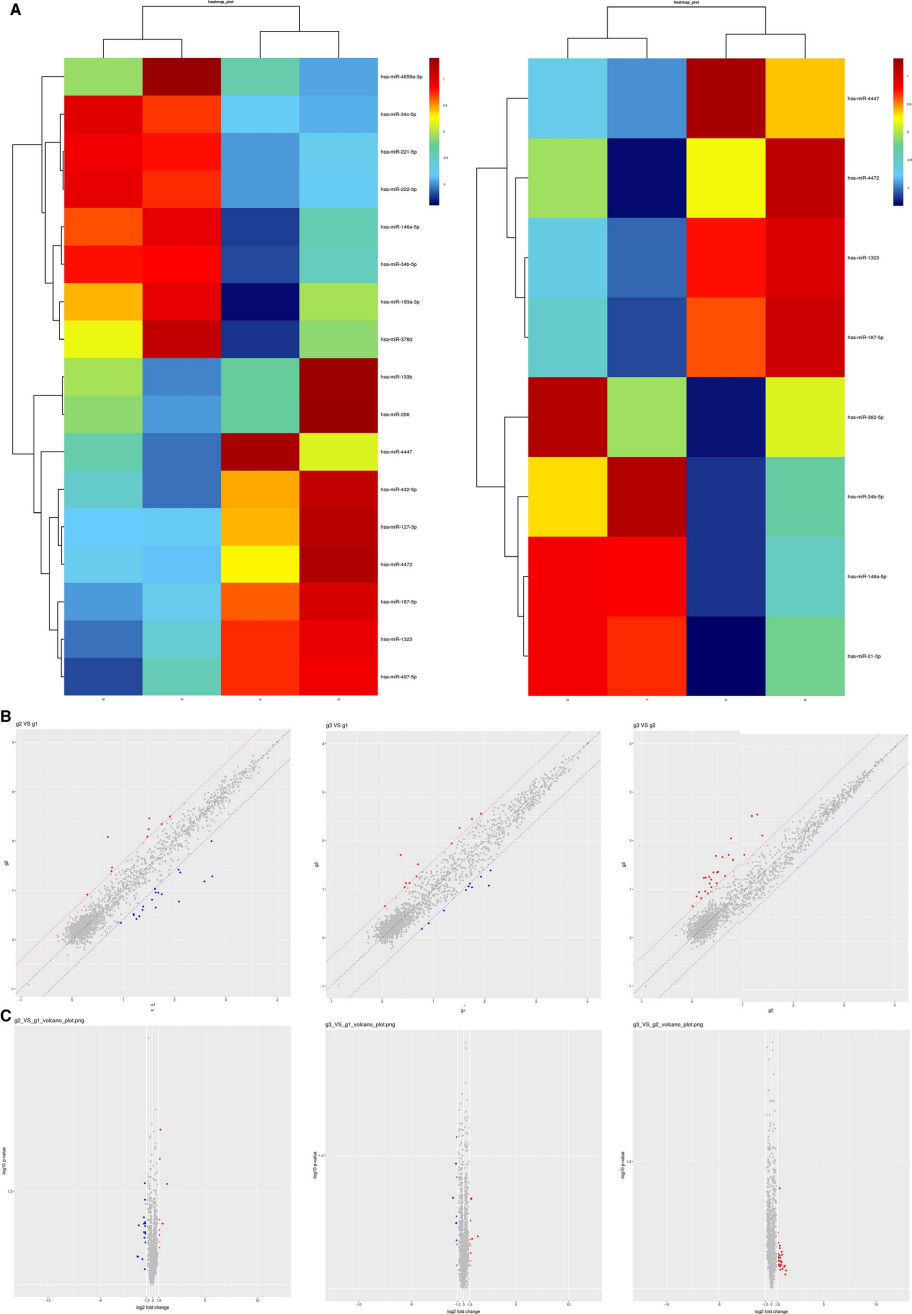
MicroRNAs were differently expressed among the three groups shown by microarrays. A, The heat map of the aberrant microRNAs (fold change [FC] > 1.5). The left showed the microRNAs between g2 and g1, while the right showed between g3 and g1. B, The scatter plots of the aberrant microRNAs (FC > 1.5). The left showed the microRNAs between g2 and g1. The middle showed between g3 and g1, while the right showed between g2 and g3. C, The volcano plots of the aberrant microRNAs (FC > 1.5). The picture sequence was similar to the aforementioned

The specimens we got during the surgeries were divided into three groups: two normal tissues adjacent to the tumors (group g1), two primary tumor tissues (group g2), and one relapsed tumor tissue + one metastatic tumor tissue in lung (group g3). The group g3 was defined that the two tumors were considered to be with higher malignancy than the tumors in group g2. These three groups' tissues were conducted with microarrays to detect the different microRNAs among them. Defining with the fold change >1.5, the heat maps (Figure 1A) showed that between g2 and g1 there were 17 different miRNAs including eight upregulated and nine downregulated. Comparing g3 with g1, there were eight different‐expressed microRNAs. However, unfortunately, few microRNAs were found between g3 and g2, so that we cannot obtain a heat map between them. Besides, three scatter plots (Figure 1B) and volcano plots (Figure 1C) were formed to exhibit the different profiles among the three groups further.

### 3.2 Functional pathways analysis of the different‐expressed microRNAs

**Figure 2:**
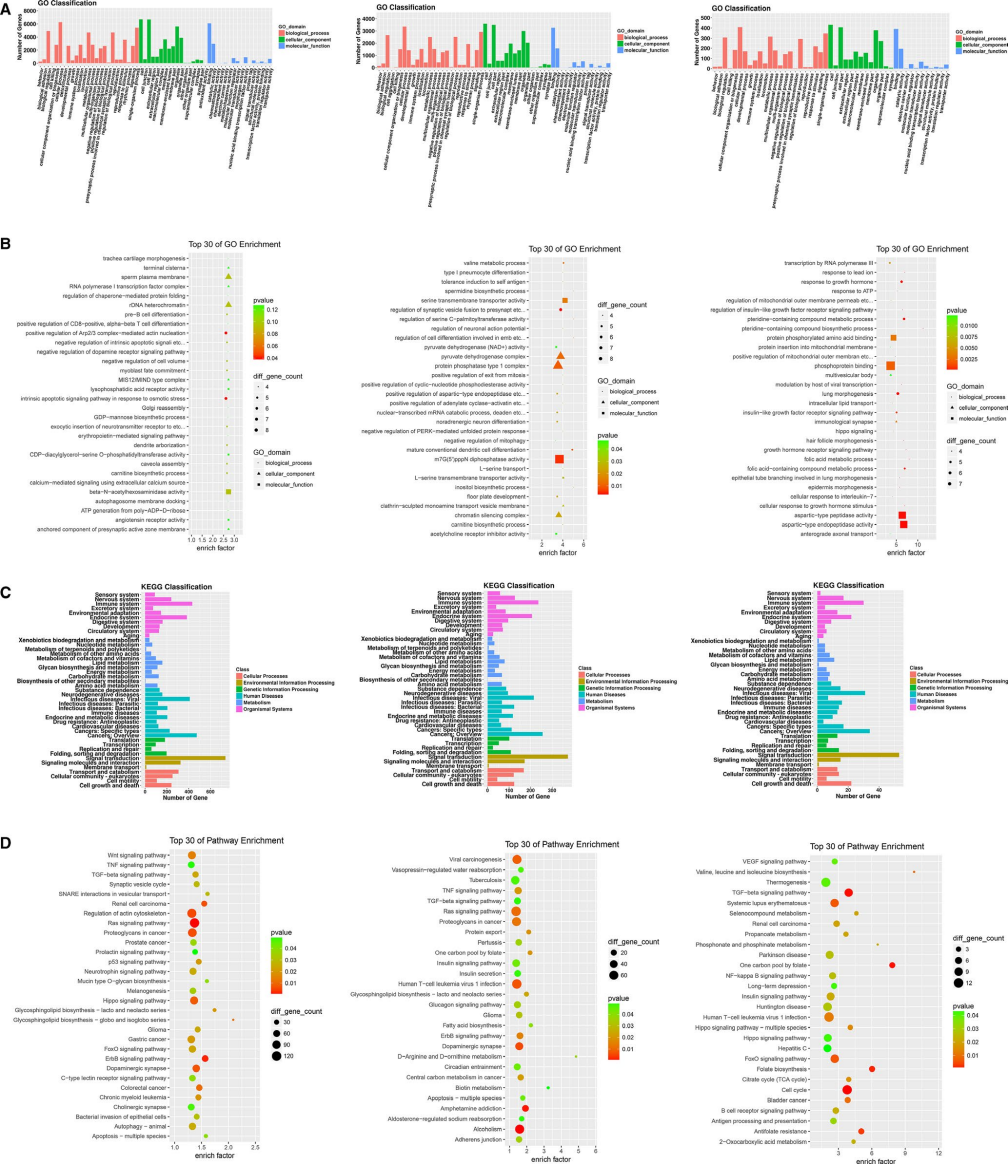
The aberrant microRNAs could have some functions. A, The results of Gene Ontology (GO) classification analysis. The left showed the compared analysis between g2 and g1. The middle showed between g3 and g1, while the right showed between g2 and g3. B, The results of GO enrichment analysis. The picture sequence was similar to the aforementioned. C, The results of Kyoto Encyclopedia of Genes and Genomes (KEGG) classification analysis. The picture sequence was similar to the aforementioned. D, The results of KEGG enrichment analysis. The picture sequence was similar to the aforementioned

Gene Ontology analysis is the classification of gene functions, including biological process, cellular component, and molecular function.^13^ We performed GO analysis among the three groups. The GO classification (Figure 2A) shows that among the three comparisons, most host genes of microRNAs were involved in cellular process of the biological process, cell part of the cellular component, and binding of the molecular function. Moreover, the GO enrichment (Figure 2B) could further elucidate that between g2 and g1, most host genes of different‐expressed microRNAs were involved with mediated actin nucleation and intrinsic apoptotic signaling pathway in response to osmotic stress. While comparing g3 and g1, most host genes were connected with the regulation of synaptic vesicle fusion to presynapt and m7G (5′) pppN diphosphatase activity. Between g2 and g3, lung morphogenesis and type endopeptidase activity were obviously involved with more host genes.

Kyoto Encyclopedia of Genes and Genomes analysis is the biological pathway classification entry.^14^ The host genes of the microRNAs mainly participated in signal transduction, immune system, and cancers according to the KEGG classification analysis of three comparisons (Figure 2C). Meanwhile, the KEGG enrichment analysis (Figure 2D) showed these host genes could be involved in the Ras signaling pathway, alcoholism, and TGF‐beta signaling pathway.

### 3.3 miR‐187‐5p and miR‐1323 were downregulated in tissues of GCTB

**Figure 3:**
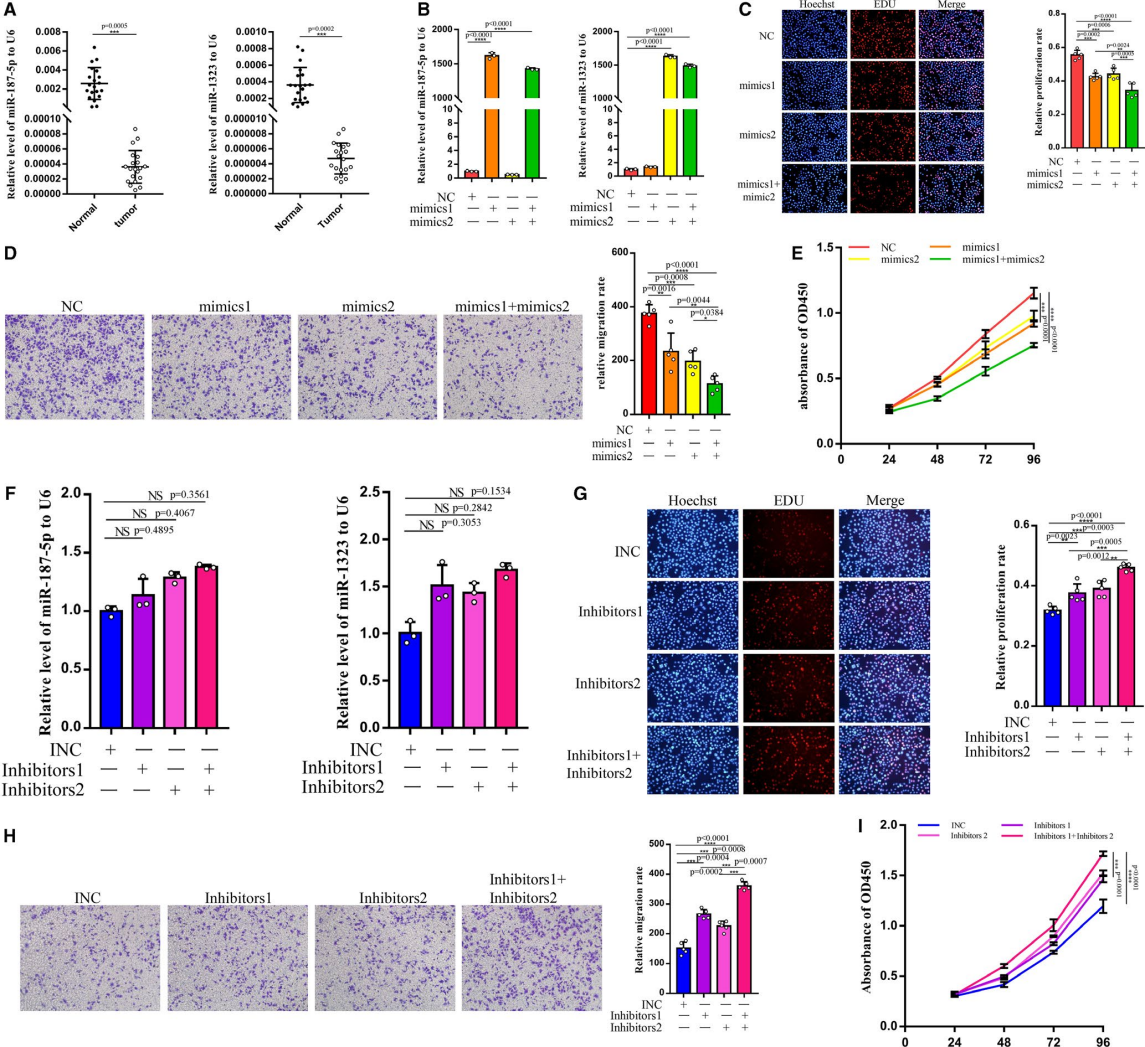
miR‐187‐5p and miR‐1323 can regulate the biological functions of GCTBSC. A, The two miRNAs had a low expression in tumor tissues shown by qRT‐PCR. B, The relative levels of the two miRNAs after the transfection of the mimics. C, After the transfection of mimics, the EDU assay examined the proliferation of GCTBSC. And, the right picture showed the statistical results. D, Transfection was tested by transwell assay and statistical result showed the significance. E, The cell counting kit‐8 (CCK8) assay showed that the mimics can inhibit the cellular viability. F, The relative levels of the two miRNAs after the inhibitors transfection. G, The EDU assay and statistic results showed that the two inhibitors can prompt the proliferation. H, The transwell assay verified the improvement of translocation. I, The CCK8 showed the change in cellular viability. “NS”, “*”, “**”, “***”, and “****” represented none sense, *P* < .05, *P* < .01, *P* < .001, and *P* < .0001, respectively. “NC”, “mimics1”, and “mimics2” represented negative control, miR‐187‐5p mimics, and miR‐1323 mimics, respectively. “INC”, “inhibitors1”, and “inhibitors2” represented inhibitors negative control, miR‐187‐5p inhibitors, and miR‐1323 inhibitors, respectively. GCTB, giant cell tumor of bone

In terms of the microarrays analysis, we found that miR‐187‐5p and miR‐1323 had low expression in tumor tissues, no matter in the primary tumors or the tumors with higher malignancy. Thus, we performed qRT‐PCR on the 20 pairs of tissues of patients with GCTB. As the results showed, miR‐187‐5p and miR‐1323 were overtly downregulated in tumor tissues (Figure 3A).

As a result, we considered that the two microRNAs may function in GCTB. To examine these microRNAs' activities in GCTB, we, respectively, transfected the microRNA mimics into the cells cultured by the GCTB tissues (GCTBSC). qRT‐PCR (Figure 3B) showed that after transfection of mimics, the relative level of miR‐187‐5p and miR‐1323 increased concomitantly. And, EDU assay was conducted. As the results (Figure 3C) showed, miR‐187‐5p and miR‐1323 mimics can separately inhibit the proliferation rate of GCTBSC, but co‐transfection of the two microRNAs can have joint influence on GCTBSC. Meanwhile, transwell assay (Figure 3D) confirmed the inhibition of translocation of GCTBSC by the two microRNAs. And, CCK8 assay (Figure 3E) was performed to verify the cellular viability. With high expression of the two microRNAs, viability of GCTBSC was overtly impaired and the situation deteriorated when co‐transfection. To further examine the effect brought by the two microRNAs, the miR‐187‐5p and miR‐1323 inhibitors were utilized and the abovementioned three function assays (Figure 3f‐i) were performed. As we expected, inhibitors of the two microRNAs had positive influence on GCTBSC.

To sum up, we can control the relative level of miR‐187‐5p and miR‐1323 to regulate the total biological functions of GCTBSC. Besides, simultaneously changing the level of the two microRNAs could have a united effect.

### 3.4 miR‐187‐5p and miR‐1323 may depress FRSC through RISC

**Figure 4:**
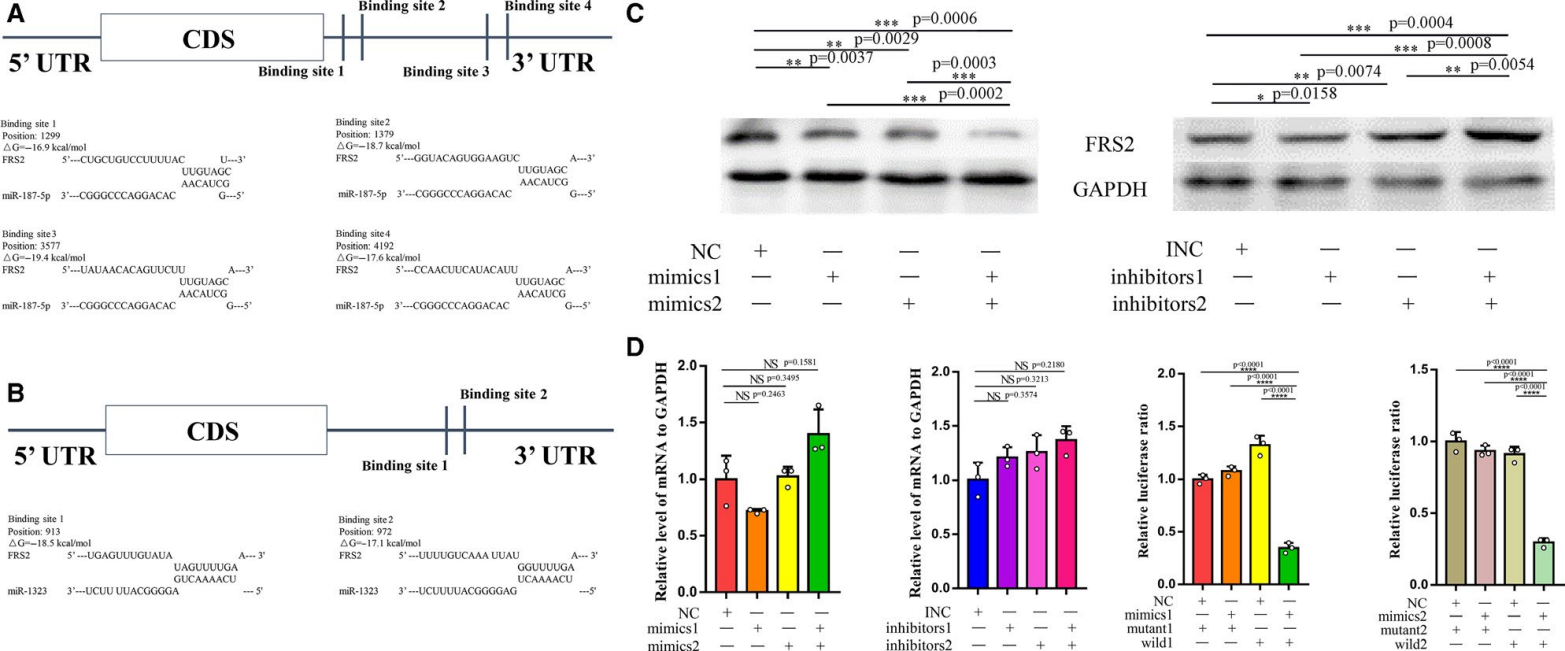
FRS2 was predicted to be targeted by miR‐187‐5p and miR‐1323 by three software. A, The schematic diagram of the binding sites of miR‐187‐5p and the binding sequence. B, The schematic diagram of the binding sites of miR‐1323 and the binding sequence. C, Western blot results that the mimics and inhibitors can regulate the protein levels of FRS2 in cells. D, The relative levels of mRNA of FRS2 in cells after the transfection of the mimics and inhibitors showed little change. E, Luciferase reporter assays confirmed the 3′UTR of mRNA of FRS2 can be bound by the two mimics, and the luciferase activities had a significant decrease while co‐transfecting with the wild and mimics. “NS”, “*”, “**”, “***”, and “****” represent none sense, *P* < .05, *P* < .01, *P* < .001, and *P* < .0001, respectively. “wild1” and “mutant1” represented the luciferase reporter plasmids that contained the wild type and mutant type of the binding sites that miR‐187‐5p could bind to the mRNA 3′UTR of FRS2, respectively. “wild2” and “mutant2” represented the luciferase reporter plasmids contained the wild type and mutant type of the binding sites that miR‐1323 could bind to the mRNA 3′UTR of FRS2, respectively

According to previous researches, microRNAs were cleaved by Drosha and Dicer, and one strand of the two was selected to bind to Argonaute proteins. RISC was formed to recognize certain mRNA, bind to it, and repress its translation. In view of the inhibited activity of miR‐187‐5p and miR‐1323, we presumed that the two miRNAs may silence some mRNAs to approach their goals. Thus, with the prediction of the software including Targetscan, miRanda, and RNAhybrid, we found that miR‐187‐5p and miR‐1323 could both bind to the 3′UTR of FRS2. As Targetscan showed 3′UTR FRS2 had four binding sites for miR‐187‐5p (Figure 4A) and two binding sites for miR‐1323 (Figure 4B). Then, with the examination of Western blot (Figure 4C), we found that mimics and inhibitors of the two microRNA can obviously alter the relative protein level of FRS2. Yet, the mRNA levels of FRS2 were relatively steady (Figure 4D). Subsequently, we assigned the 3′UTR of FRS2 into the luciferase reporter vectors (wild), and also the mutant of the 3′UTR (mutant) was implemented as control. After the co‐transfection of two mimics with the wild and mutant luciferase reporter vectors separately, the mimics could evidently decrease the luciferase activities in GCTBSC, while mutant vectors had little influence on the luciferase activities (Figure 4E).

As a result, we determined that miR‐187‐5p and miR‐1323 could repress the expression of FRS2 through the binding of the 3′UTR of mRNA.

### 3.5 Inhibition of FRS2 could limit the biological functions of GCTBSC

**Figure 5:**
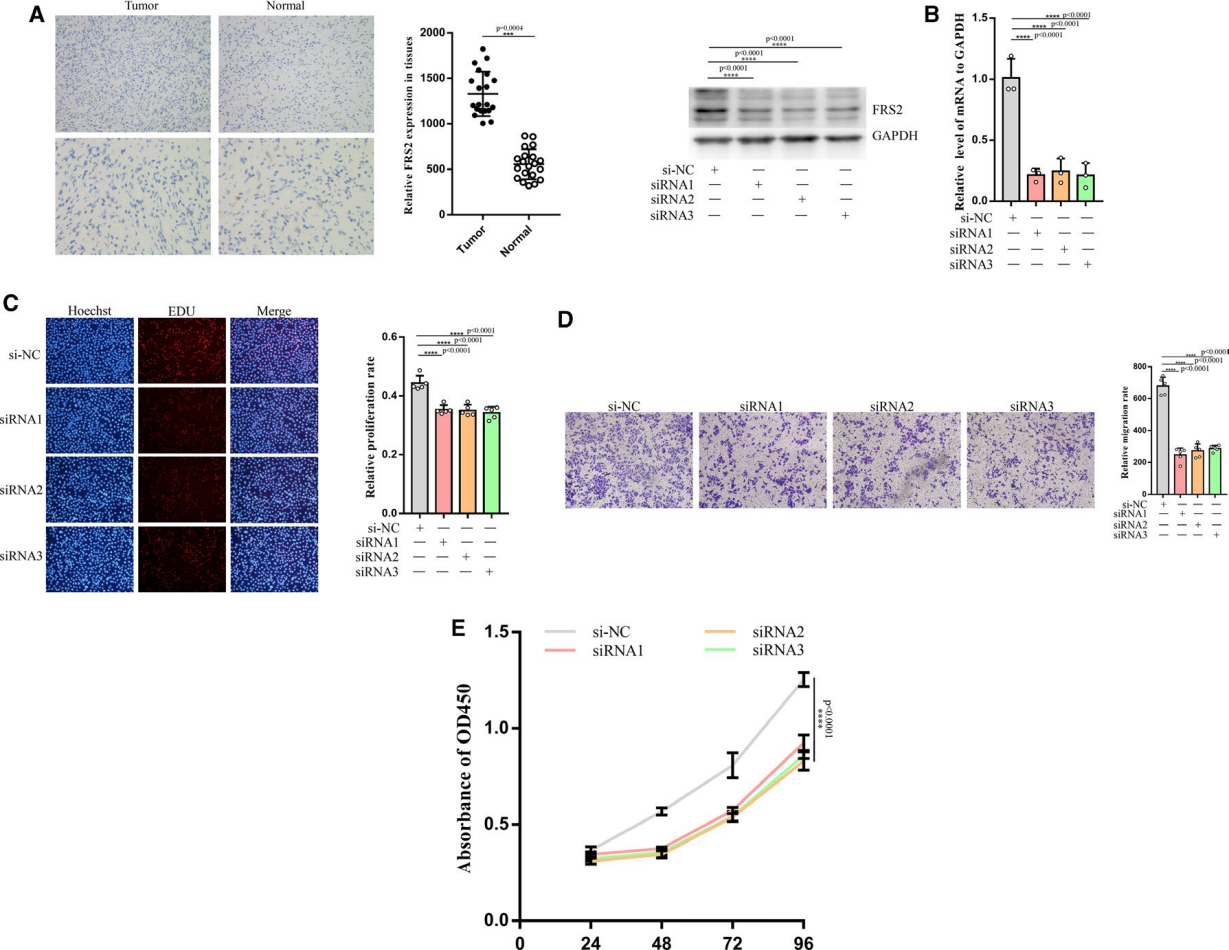
FRS2 was downregulated in tumor tissues and can influence the functions of GCTBSC. A, The immunohistochemistry of FRS2 in tumor tissues and normal tissues. The above two photos were taken at 200× and the down were taken at 400×. And, the statistical result showed the significance. B, After transfection of three siRNAs and negative control, the relative protein and mRNA levels of FRS2 in cells were examined by Western blot (left) and qRT‐PCR (right). C, The EDU assay and its statistical result showed that the three siRNAs can overtly block cell proliferation. D, The transwell assay and its statistical result showed the inhibition of translocation by the three siRNAs. E, Cellular viability was tested by CCK8 and downregulated. “***” and “****” represent none sense, *P* < .05, *P* < .01, *P* < .001, and *P* < .0001, respectively. “si‐NC” represented siRNA negative control. GCTB, giant cell tumor of bone

Although we had elucidated the binding of miR‐187‐5p and miR‐1323 to the 3′UTR of FRS2, the functions of FRS2 in GCTB have yet been ambiguous. Firstly, we detected the expression of FRS2 in tumor tissues and normal tissues of patients with GCTB. And, the relative protein level was probed by immunohistochemistry. As the result (Figure 5A) showed, FRS2 had an ectopic expression in GCTB. To further dig into its functions in GCTB, we designed three types of siRNAs targeting FRS2 (siRNA1, siRNA2, siRNA3 sequences shown in Table S2). And, the three siRNAs can evidently decrease the protein and mRNA level of FRS2 in GCTBSC demonstrated by Western blot and qRT‐PCR (Figure 5B). Following on the transfection of the siRNAs, aforementioned function assays (Figure 5C‐E) were conducted in GCTBSC. As expected, siRNAs targeting FRS2 had generated significant suppressive impact on GCTBSC. And, the functions of forced expression of FRS2 would be stated later. With the evidences shown above, we can conclude that FRS2 may play an important role in GCTB as a proto‐oncogene. And, the downregulation of its expression in GCTBSC could impose a restriction on tumor activities.

### 3.6 miR‐187‐5p and miR‐1323 can regulate biological functions of GCTB through the repression of FRS2

**Figure 6:**
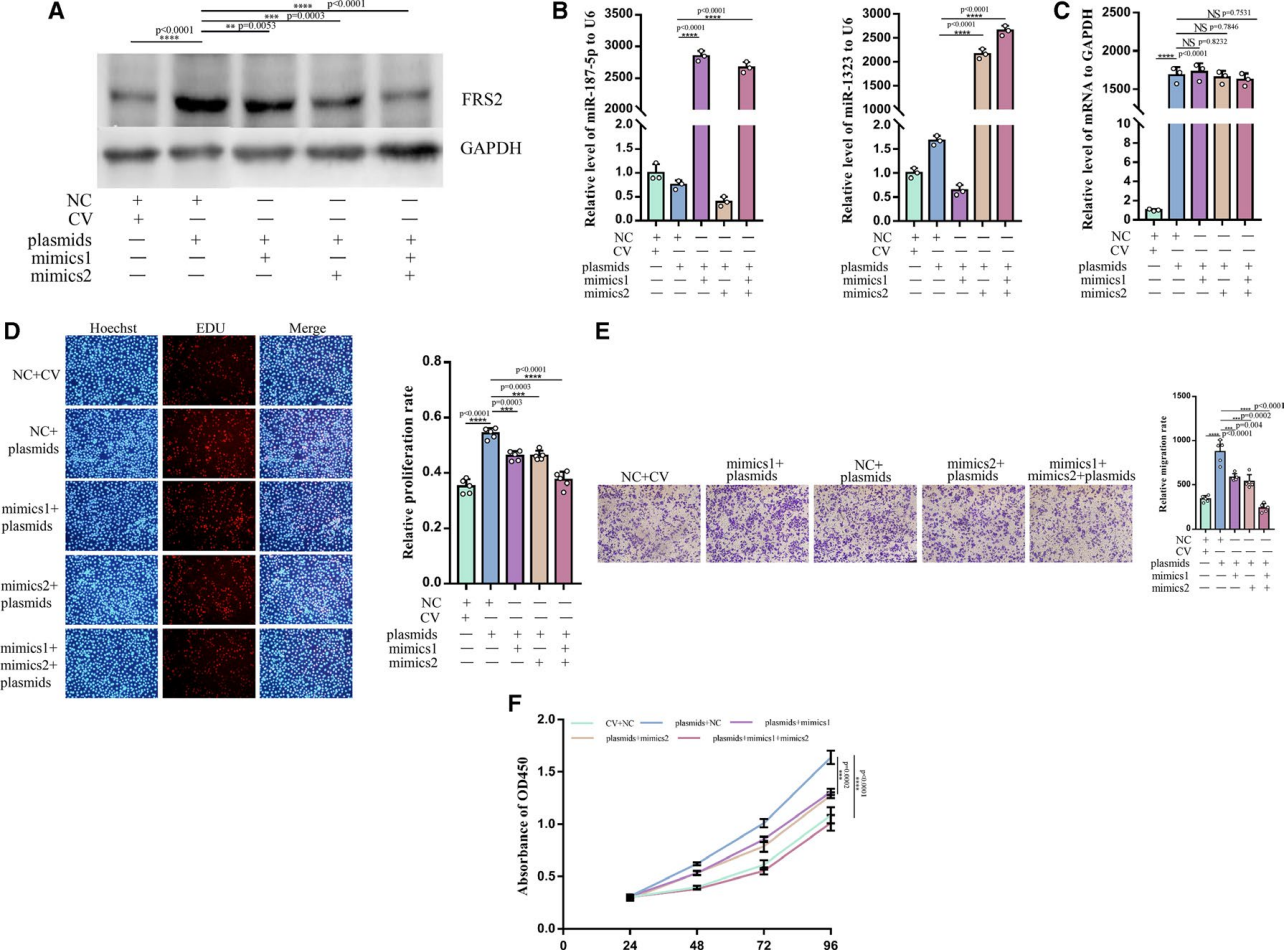
miR‐187‐5p and miR‐1323 can regulate the biological functions of GCTB through blocking the expression of FRS2. A, After the co‐transfection, the relative protein levels of FRS2 in cells. B, qRT‐PCR showed the relative miRNA levels after co‐transfection. C, The relative mRNA levels of FRS2 increased after adding FRS2‐overexpressed plasmids. D‐F, The function experiments showed the mimics can rescue the influence exerted by the plasmids. “NS”, “**”, “***”, and “****” represent none sense, *P* < .05, *P* < .01, *P* < .001, and *P* < .0001, respectively. “CV” and “plasmids” represented the negative vectors and FRS2‐overexpressed plasmids, respectively. GCTB, giant cell tumor of bone

The functions of miR‐187‐5p and miR‐1323 had been proved in GCTBSC, as well as FRS2. However, we could not figure out the exact mechanism of the influence of the two microRNAs exerting on GCTBSC. To prove the conjecture that the two microRNAs realized their roles by silencing FRS2 in GCTBSC, we performed the rescue experiments. The vectors forcedly expressing FRS2 were designed and co‐transfected with the two microRNA mimics into GCTBSC. As Western blot (Figure 6A) showed, forced expression of FRS2 could be recovered to the level of negative control by the addition of the two microRNAs. Nonetheless, the relative mRNA level of FRS2 had little change even through the interruption of the two microRNAs (Figure 6B,C). Far more importantly, as far as the function assays (Figure 6D‐F) conducted, the FRS2‐overexpressed plasmids could prompt the total cellular activities, including proliferation, translocation, and cellular viability. Yet, after the addition of the two kinds of mimics, the positive influence could be alleviated, especially the combined addition of miR‐187‐5p and miR‐1323 mimics.

As a result, we had a conclusion that the co‐transfection of the FRS2‐overexpressed plasmids and microRNA mimics could adjust the influence caused by the sole transfection of FRS2‐overexpressed plasmids. Further, we could also reach that miR‐187‐5p and miR‐1323 could regulate the biological functions of GCTB through repressing the expression of FRS2.

## 4 DISCUSSION

Giant cell tumor of bone is a borderline tumor, which has a high recurrence and a tendency to translocation, especially the lung.^1^, ^2^ Even though Denosumab has been approved to treat for GCTB,^5^ there exists a need to demonstrate the mechanism of occurrence and development of the tumor in order to find more effective molecules functioning in GCTB.

MicroRNAs are considered as one kind of small noncoding RNAs, with a length of 19‐25 nt.^6^ It is recognized that microRNAs could form into RISC with Argonaute proteins and repress the translation of certain mRNAs.^7^ There have been some research on microRNAs in GCTB. Quan Huang's team found that miR‐30a can target RUNX2 to inhibit the osteolysis in GCTB.^11^ And, Ingrid Herr's research proved that miR‐127 and miR‐376a can act as tumor suppressors through the silence of COA1 and PDIA6 in GCTB.^12^ Nonetheless, few researches on microarrays conducted in GCTB have been launched. We performed the microarrays on three groups of GCTB and found two microRNAs, miR‐187‐5p and miR‐1323, downregulated in tumor tissues, no matter primary tissues or recrudescent tissues. miR‐187‐5p has been found to target CYP1B1 to suppress cancer cell progression in non‐small cell lung cancer.^15^ Besides, Li et al proved that miR‐187‐5p may be associated with cell biological functions of bladder cancer.^16^ It may be involved with drug sensitivity in breast cancer cell lines as well. Initially, miR‐1323 was raised to be upregulated in complete hydatidiform moles.^17^ And then, Priscilla T‐Y Law's team predicted that miR‐1323 was upregulated and linked with the poor prognosis in hepatocellular carcinoma.^18^ Afterward, it has been found in the prediction of resistance to neoadjuvant radiochemotherapy in squamous cell carcinoma of the esophagus.^19^ However, there has no research exploring their functions in GCTB. Thus, our team firstly discovered the aberrant expression of the two microRNAs and examined their functions in GCTB.

miR‐187‐5p and miR‐1323 have been examined to synergistically regulate the biological functions of GCTB in vitro as we utilized the mimics and inhibitors. Yet, to elucidate the deep mechanism behind the regulation, we conjectured that miR‐187‐5p and miR‐1323 may have a common targeted mRNA, FRS2, through the calculation and prediction of the three software. Fortunately, after the transfection of the two microRNA mimics and inhibitors, the protein level of FRS2 had some predicted changes with the stable level of mRNA. Besides, luciferase reporter assays further confirmed our forecast. Fibroblast growth factor receptor (FGFR) substrate 2 is one type of the adaptor/scaffold protein, which can bind to receptor tyrosine kinases and activate downstream signals.^20^ Zhang et al found that FRS2 had a high expression in high‐grade liposarcoma and activation of the FGFR/FRS2 signaling may play an important role in the development of liposarcoma.^21^ Dey's team launched a research that inhibition of FRS2 could block the PI3K/AKT signaling to induce apoptosis and suppress the proliferation and translocation in breast cancer.^22^ Song Wu et al figured out that FRS2 had an obviously upregulation in bladder cancer through whole‐genome sequencing, which could recruit endothelial cells and induce tube formation.^23^ Based on these studies, we conducted immunohistochemistry in GCTB tissues and adjacent normal tissues, and found that FRS2 was highly expressed in tumor tissues. Besides, siRNA targeting FRS2 confirmed that interruption of its expression in GCTBSC could alleviate the tumor activities. Finally, we co‐transfected plasmids expressing FRS2 with the two kinds of mimics into GCTBSC to verify that the plasmids can exacerbate the tumor activities, while the addition of the two mimics could recover the influence of the plasmids.

Unfortunately, we could not construct the animal models of GCTB so that we have not examined our prediction in vivo. Even though there are some researches mocking the tumors with the chick embryo chorioallantoic membrane assay,^24^ we have been doubting its reliability and validity.

## 5 CONCLUSION

Through microarray, we found the ectopic expression of several microRNAs in GCTB. miR‐187‐5p and miR‐1323 were highly expressed in tumor tissues, which could unitedly regulate biological functions of GCTB in vitro. Furthermore, mRNA of FRS2 could be targeted and repressed by the two microRNAs, so as to compromise the tumor activities.
