# Supplementary figures and images for: Expression profiles of miRNAs in giant cell tumor of bone showed miR‐187‐5p and miR‐1323 can regulate biological functions through inhibiting FRS2
Source: Cancer Med. 2020 Mar 10;9(9):3163–73. doi: 10.1002/cam4.2853 (PMC7196053; doi:10.1002/cam4.2853)

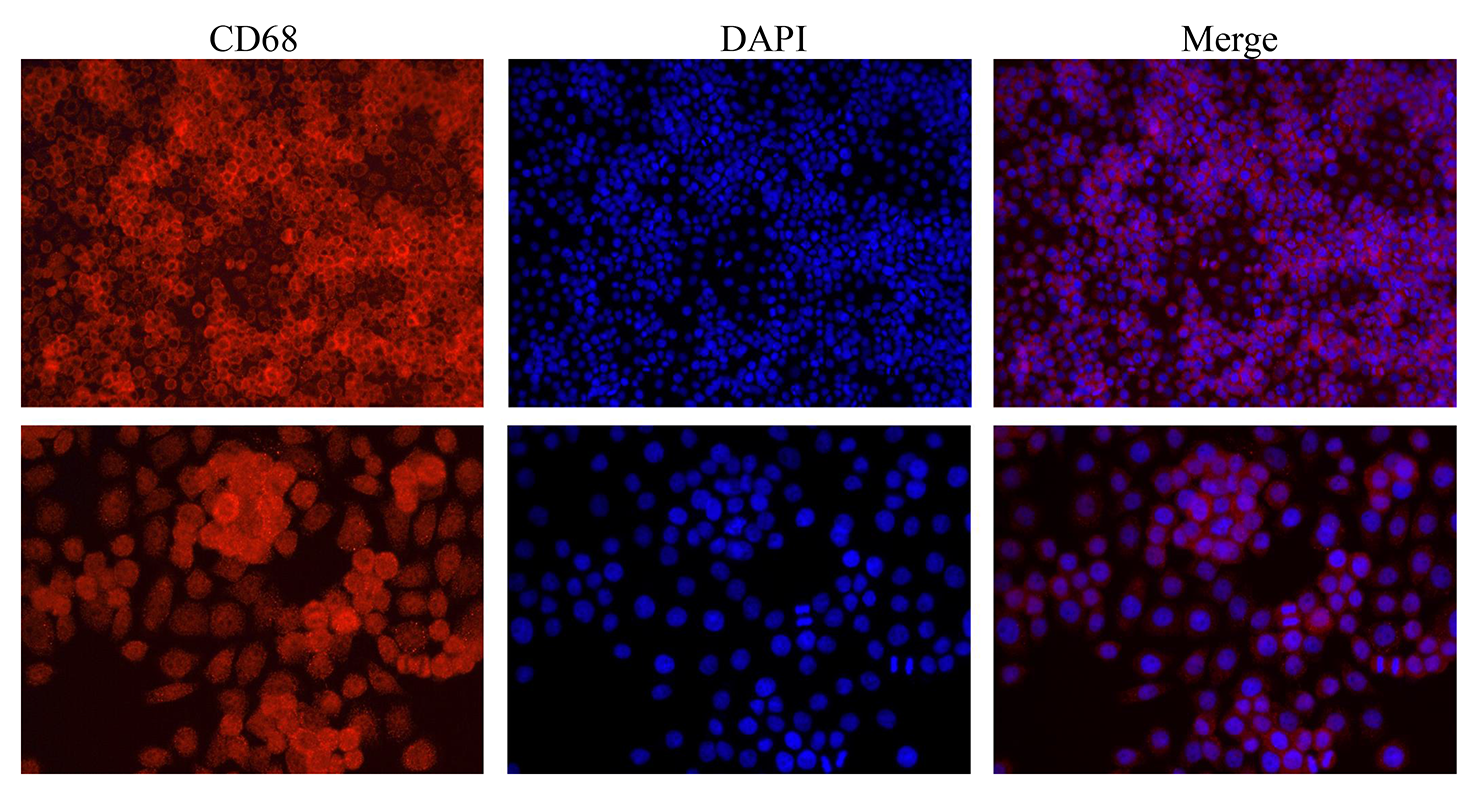

Supplement: Supplementary file 1 [file CAM4-9-3163-s001.tif]

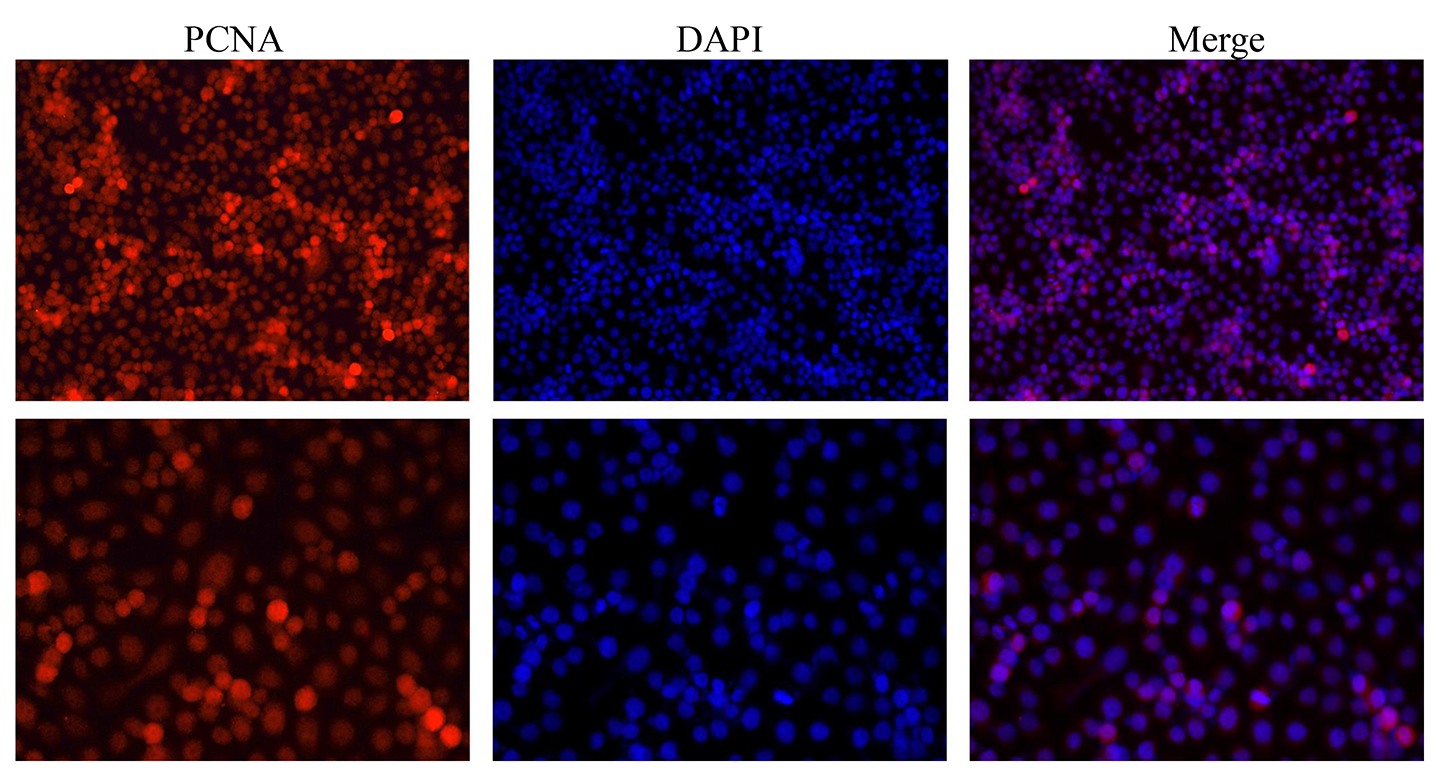

Supplement: Supplementary file 2 [file CAM4-9-3163-s002.tif]

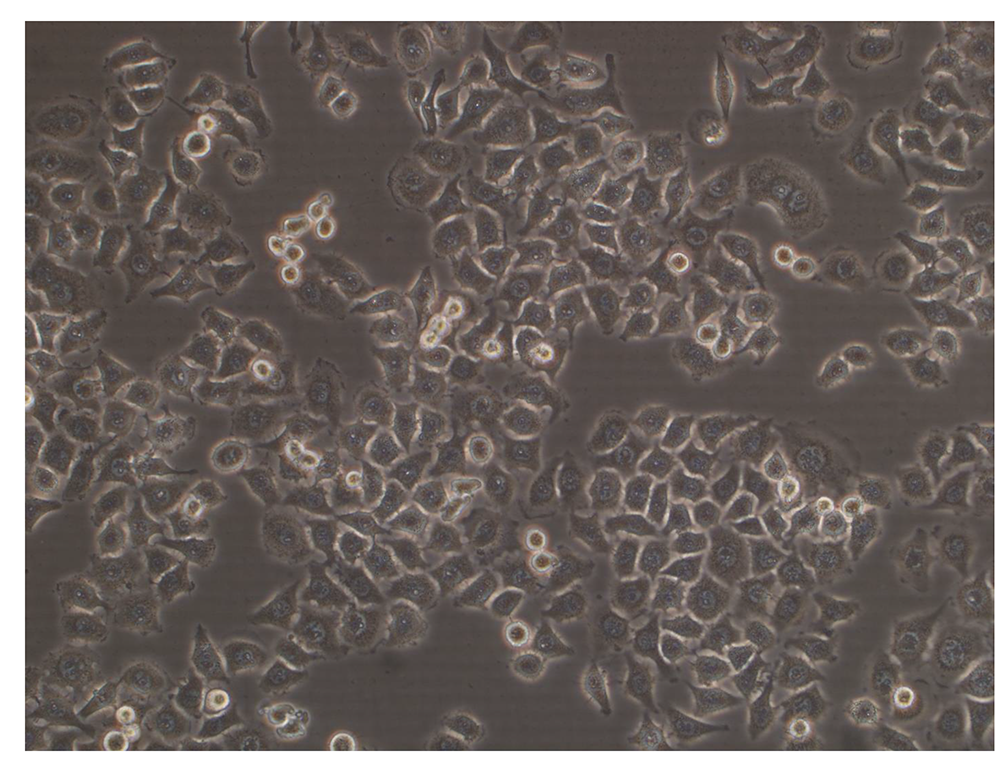

Supplement: Supplementary file 3 [file CAM4-9-3163-s003.tif]
