# Supplementary material for: Expression profiles of miRNAs in giant cell tumor of bone showed miR‐187‐5p and miR‐1323 can regulate biological functions through inhibiting FRS2
Source: Cancer Med. 2020 Mar 10;9(9):3163–73. doi: 10.1002/cam4.2853 (PMC7196053; doi:10.1002/cam4.2853)
Supplement: Supplementary file 4 [file CAM4-9-3163-s004.docx]

**
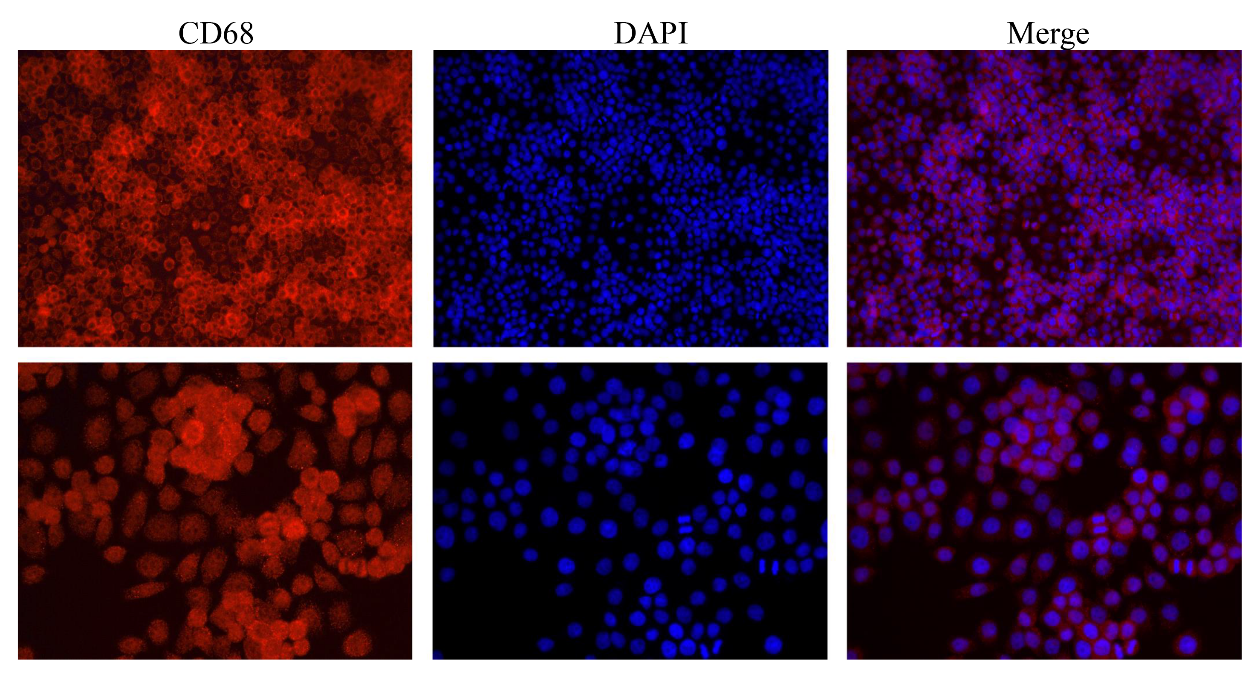
Supplementary**

S1: the immunofluorescence level of CD68 in 0404 cell line. The photos upside were taken at 200X, while the photos downside were taken at 400X. The red fluorescence represents CD68 and the blue represents nucleus.


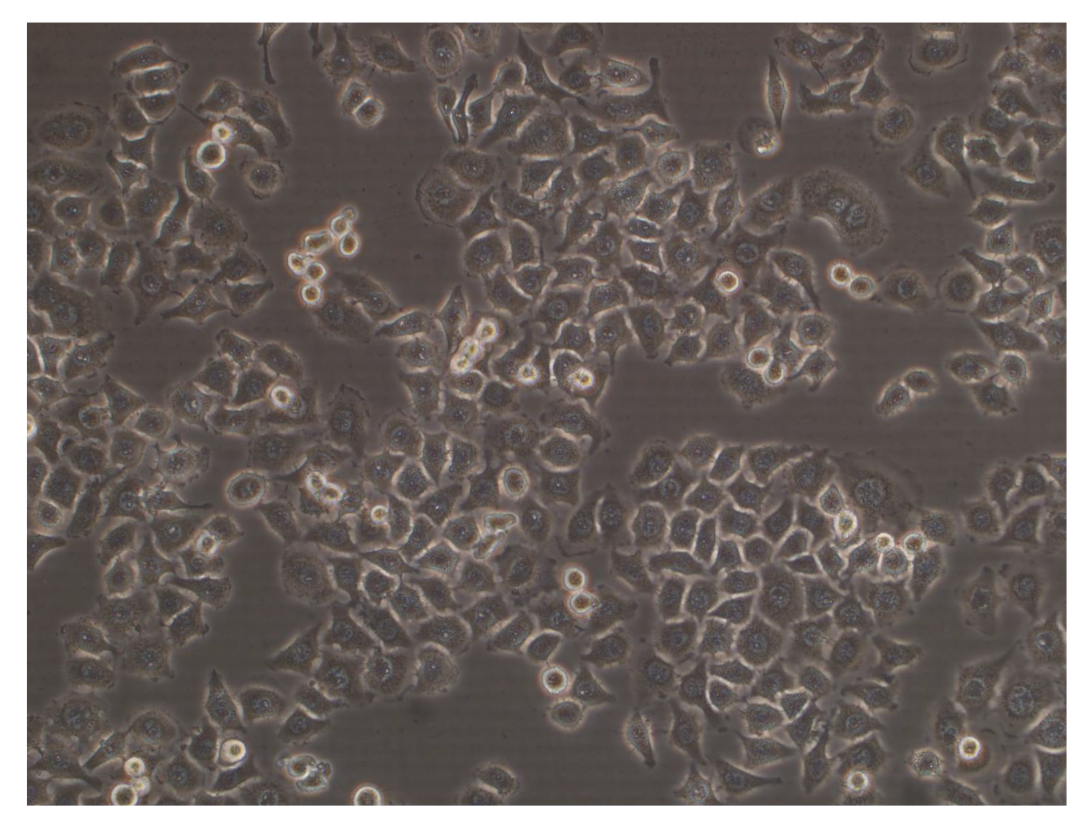

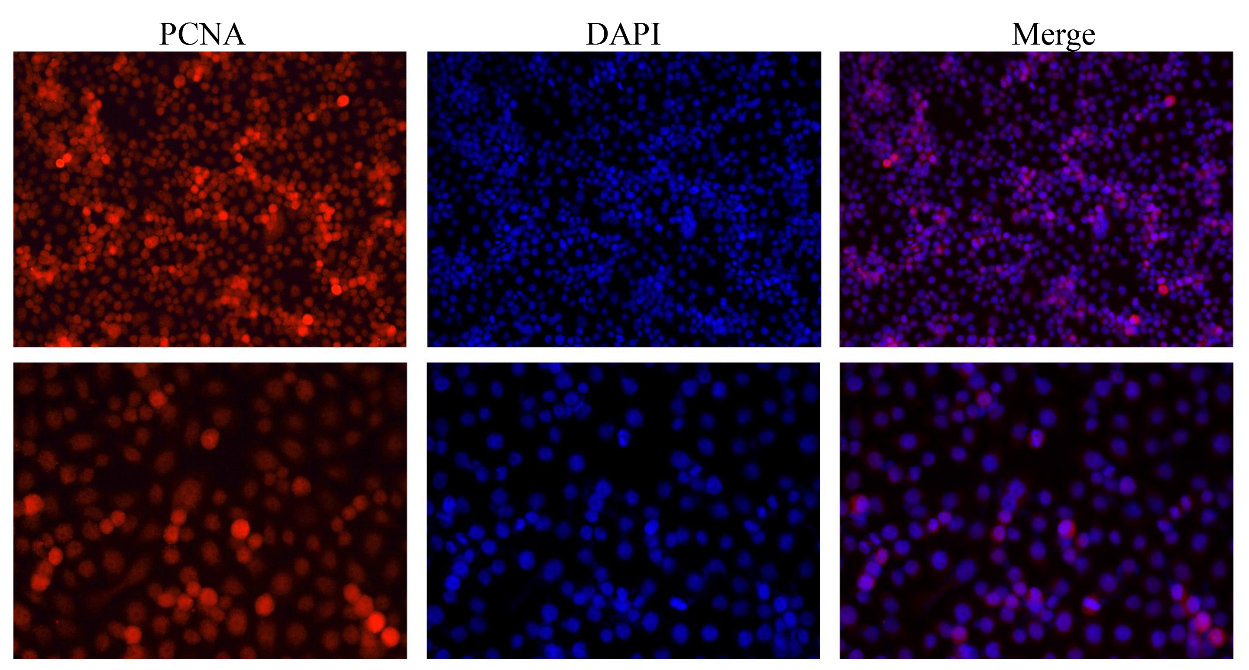
S2: The immunofluorescence level of PCNA in 0404 cell line. The photos upside were taken at 200X, while the photos downside were taken at 400X. The red fluorescence represents PCNA and the blue represents nucleus.

S3: the morphology of 0404 cell line is shown. The photo was taken at 200X. We can see that cells grew with colony, and mainly consist of spindle cells with a few of mononuclear circle cells and multinucleated giant cells.

**Table 1** the general information of patients

| Number | Gender | Age（year） | Tumor location |
| --- | --- | --- | --- |
| Patient 1 | male | 17 | proximal left tibia |
| Patient 2 | female | 29 | distal left femur |
| Patient 3 | female | 46 | distal left femur |
| Patient 4  Patient 5  Patient 6  Patient 7  Patient 8  Patient 9  Patient 10  Patient 11  Patient 12  Patient 13  Patient 14  Patient 15  Patient 16  Patient 17  Patient 18  Patient 19  Patient 20 | male  male  female  male  female  female  male  female  female  male  female  male  male  female  female  male  female | 33  35  22  62  25  34  31  59  43  46  19  25  33  35  41  60  42 | proximal left tibia  proximal left tibia  proximal right tibia  distal left humerus  distal left femur  distal right femur  proximal left tibia  distal right radius  distal left radius  distal left humerus  distal right humerus  distal left femur  distal right femur  proximal left tibia  distal right femur  proximal left tibia  distal right femur |

**Table 2 the sequences of primers and siRNAs**

|  | Sequence |
| --- | --- |
| FRS2 primer | F: AGTGCCTCAGGGGTCAGGAG  R: CGGGCTTCCCAAACAGGAGG |
| si-NC/NC | 5’UUCUCCGAACGUGUCACGUTT  3’TT AAGAGGCUUGCACAGUGCA |
| siRNA1 (FRS2α-home-934) | 5’GCUGCUCAGAACUUACCUATT  3’TT CGACGAGUCUUGAAUGGAU |
| siRNA2 (FRS2α-home-993) | 5’ CCAUCCGUCAAGCAGACAUTT  3’TT GGUAGGCAGUUCGUCUGUA |
| siRNA3 (FRS2α-home-1978) | 5’ CCACGAGAUGAUGGUACAUTT  3’TT GGUGCUCUACUACCAUGUA |
